# Homogeneity of Sand-Sized Microplastics Concentration and Polymer Assemblage in Beach and Coastal Dune Sediments

**DOI:** 10.1021/acs.est.5c09174

**Published:** 2025-10-28

**Authors:** Rasma Ormane, Andreas CW Baas

**Affiliations:** † Department of Geography, 4616 King’s College London, 30 Aldwych, London WC2B 4BG, United Kingdom

**Keywords:** enrichment, composition, diversity, supply-limited

## Abstract

Microplastics impact marine and terrestrial environments as vectors of chemical pollution, stunting the growth of dune plants and posing a threat to coastal ecosystems. Because they are more easily transported by wind from the beach environment into the coastal dunes than mineral sand grains, dune sediment ought to be relatively enriched in microplastics. To test this hypothesis, the concentrations and polymer assemblage of sand-sized (0.1–1 mm) microplastics (MPs) in surface sediment were compared between intertidal beach (marine) and coastal dune (aeolian) samples at two different UK coasts (mid-Wales adjacent to the Irish Sea and southeast England adjacent to the English Channel) using FT-IR microscopy. Results show no differences in polymer composition, diversity, or abundance between beach and dune sediments. Average concentrations reached hundreds of MPs/kg, and their composition was dominated by rayon and polyester fibers. The lack of expected microplastics enrichment of the coastal dunes by preferential aeolian transport from the adjacent beach is attributed to the severe supply limitation of these particles at the sediment surface interface compared with the transport-limited movement of wind-blown mineral sand. Our findings suggest that mitigation of microplastics contamination may focus on the beach environment, as its condition is mirrored in the dune sediment.

## Graphical abstract

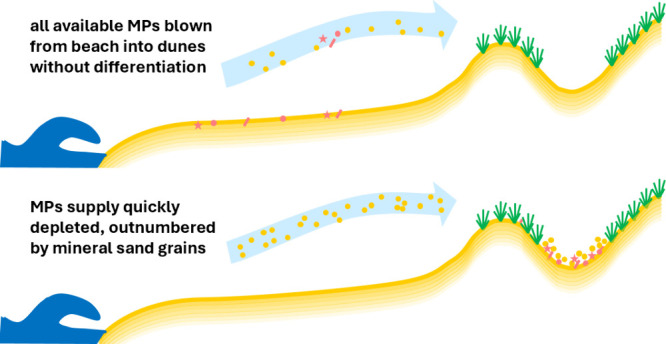

## 1 Introduction

Microplastics are recognized as a globally ubiquitous contaminant found in all marine and terrestrial environments, from isolated islands in the South Pacific to the top of Mount Everest.^1^ Generally defined as particles ranging from 1 μm to 5 mm in diameter consisting of various synthetic polymers, microplastics (here onward abbreviated “MPs”) are suspected to pose a health risk to humans,^2,3^ and they impact all biotic food chains and ecosystems as vectors of harmful microbes, chemical pollutants, and radioactive nuclides.^4–6^ Studies of the atmospheric limb of the MPs cycle have revealed its impact on air quality and soil contamination,^7^ with sea spray of marine MPs and entrainment of MPs from agricultural fields conjectured to be key sources of atmospherically transported particles.^8,9^

Coastal environments such as beaches and dunes have so far not been considered as sources, nor has there been any direct field evidence on the wind-blown transport of MPs from intertidal beaches into coastal dunes, even though this constitutes a key interface between the marine reservoir and terrestrial ecosystems. Through their leached degradation products, MPs contamination in coastal dunes has been shown to reduce seed germination, stunt early plant growth, and pose a threat to coastal ecosystems.^10^ The current assumption is that coastal dunes accumulate MPs blowing in from the intertidal beach, similar to the accumulation of meso- and macroplastic litter,^11,12^ because MPs are thought to be more easily transported by wind off the beach and into the coastal dunes than mineral sand grains.

There is, however, little understanding of potential wind-blown transport of sand-sized MPs among mineral sediment in aeolian environments. A 2019 study in Iran^13^ measured the preferential transport of MPs in wind-blown sediments over 11 agricultural fields in a portable wind tunnel. Results showed airborne sediment having MPs concentrations of 20 mg/kg from an underlying soil containing only 7 mg/kg—an enrichment ratio of ∼3. Wind tunnel experiments by Bullard et al.,^14^ meanwhile, have demonstrated the importance of particle shape for the transport of MPs within active wind-blown sediment, with fibers being mobilized more easily through enrichment ratios up to ∼1000, compared with up to only ∼30 for microbeads. These wind tunnel results contrast markedly, however, with real-world measurements of MPs in wind-blown sediment caught in traps on arid agricultural fields in northern China,^15^ which show less obvious preferential transport (enrichment ratios from 0.8 to 1.15 reported by Tian et al.^15^ and from 0.22 to 1.35 for sand-sized MPs by Tian et al.).^16^

To date, there have been only two studies on MPs in coastal dunes. Costello and Ebert^17^ sampled five coastal dune locations in the Great Lakes region, USA. They found on average up to ∼10 particles per square meter and no obvious dependence on the distance from the shoreline. A more comprehensive study by Anderson and Turner^18^ found a total of 332 particles from 16 beach samples and 8 dune samples at Braunton Burrows, SW England. While their results suggested a possible relationship between MPs concentration and the very fine sand fraction, they found no difference in concentrations between beach and dune sediments. However, they did not analyze and compare the chemical composition of the particles, leaving open the possibility that certain plastic types may yet show a relative enrichment in the dune sediment.

In the study reported here we test the hypothesis of relative MPs enrichment of coastal dunes, exploring similarities and differences in not only the abundance but also the assemblage (polymer type, shape, color) of MPs between beach (marine) sediment and dune (aeolian) deposits. We sampled surface sediment along transects from the intertidal beach into coastal dunes at two different UK coasts (Ynyslas, Wales, and Camber Sands, SE England). We extracted and identified in total nearly 1000 MPs from 20 samples, and we determined the polymer composition using FT-IR spectroscopy of almost 800 of these. The study is the first of its kind to compare MPs assemblages between beach and dune environments, and it expands the number of coastal dune regions investigated globally so far from two to four.

Another innovative aspect of our work is the processing of relatively large (200 g) sediment samples for extracting MPs with a high-density separation fluid and FT-IR identification to reduce the potential error bars around scaled-up concentrations. While the challenges around comparing different MPs studies due to variability in methodological protocols and analysis are widely recognized, one key limitation not commonly highlighted is that the quantity of sediment being analyzed is often very small, on the order of 5 or 10 g. In many studies, the resulting MPs counts are subsequently scaled by a factor of 100 or 200 to report concentrations per kg of sediment. Not only does this result in very wide error bars, but the minute sample size also contributes greatly to intersample variability, as MPs are unlikely to be distributed perfectly homogeneously in the sediment to begin with. In the context of aeolian deflation of a beach surface, for example, erosion to a depth of 5 mm (not an unrealistic scenario for a single wind event) yields a volume of 5000 cm^3^, or roughly 8 kg of dry sediment per square meter of surface. A 10 g sample analyzed for MPs then constitutes only 0.125% of a representative amount of dynamic sediment in the field. In the Materials and Methods section below we describe our original lab protocol development in detail to highlight some of the specific challenges involved with the processing of sand samples at bulk scale.

## 2 Materials and Methods

### 2.1 Study Sites and Sampling

Sediment samples were collected at Ynyslas, in mid-Wales, and Camber Sands, in southeast England, both in the UK (Figure 1a), during or shortly after the season of dominant onshore sand-transporting south-westerly winds to ensure that the dune surface sediment had been freshly blown in from the beach. Ynyslas is a sandy spit backed by a substantial coastal dune system. The shore runs roughly north–south and is exposed to westerly winds and a spring tidal range of 5.0 m. Camber Sands is a sandy beach and dune system east of the River Rother mouth, running roughly east–west and experiencing a spring tidal range of 6.7 m. Camber Sands attracts significant beach tourism: in the summer of 2021, for example, it reported around 25,000 visitors per day,^19^ overall reaching more than a million tourists a year.^20^ Ynyslas, in contrast, had only just over 130,000 visitors in the whole year of 2021.^21^

**Figure 1:**
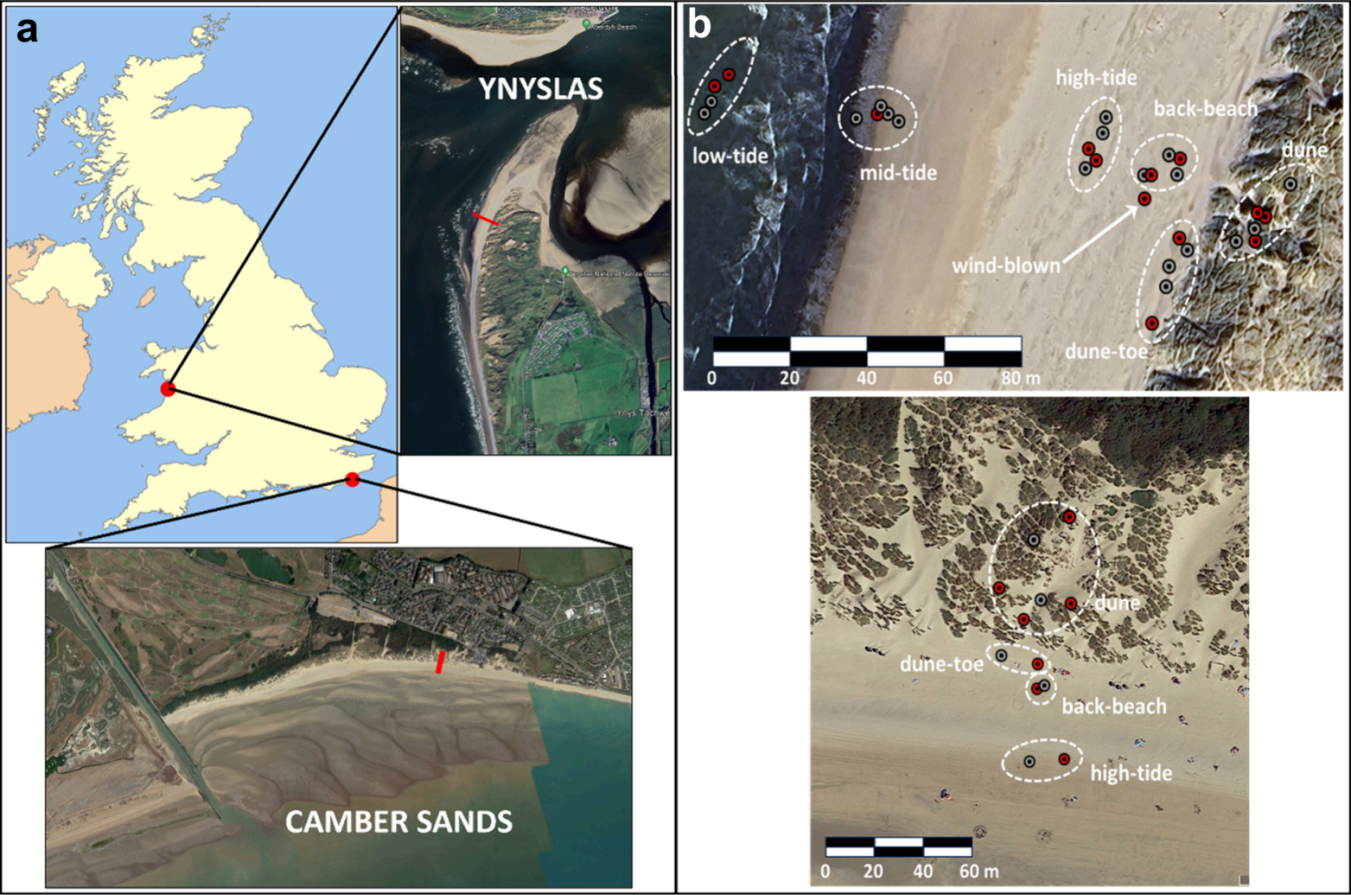
Two coastal sites sampled in the UK: Ynyslas (mid-Wales; 52.5305° N, −4.057200° W) and Camber Sands (SE England; 50.9332° N, 0.7933° E), with transects indicated by red line segments (a). Locations of surface samples at each site (b): Ynyslas, top-right; Camber Sands, bottom-right. Red symbols indicate the samples that were fully analyzed.

At Ynyslas, surface samples were collected on April 13, 2023 during low tide and a strong onshore wind, along a 160 m long transect from west to east (Figure 1b). Multiple beach surface samples were collected, within 10 m from each other, in clusters around each of the following: the low-tide mark (4 samples), halfway toward as well as at the high-tide mark (5 at each), and the back-beach (5 samples), defined here as the likely reach of the highest spring tide. The dune area was separated from the beach by a 0.5 m high step formed by wave-erosion of the dune-toe during a previous storm event. Five samples were collected from the surface above this step (“dune-toe”) and on top of the first dune row itself (5 samples). Because the site experienced active aeolian activity, we collected three samples of wind-blown sand using small Bagnold traps at the back-beach.

At Camber Sands, surface samples were collected on July 18, 2023 during low-tide and wind-still conditions, along a 100 m long transect from south to north (Figure 1b). Pairs of samples were collected at the high-tide mark, the back-beach, the dune-toe, as well as in a blowout halfway up the dune slope, together with four samples from the top of the foredune.

Each surface sample was obtained by use of a 0.5 × 0.5 m metal survey quadrat, gridded at 0.1 m spacings, placed on a section of ground unobstructed by exposed debris or pebbles. Sediment was collected using a stainless-steel spoon, scooping up material from down to a depth of ∼5 cm from 12 randomly chosen squares within the quadrat (i.e., a total sampling area of 0.12 m^2^) and storing this in a sealed aluminum foil tray for transport to the laboratory. The field samples amounted to ∼700 g of (dry-weight) sediment. This sediment sampling procedure is similar to that deployed in the two comparable coastal dune studies.^17,18^

### 2.2 Sample Preparation

Samples were oven-dried for 48 h at 45 °C to remove moisture, after which 200 (±2) g (dry-weight) was taken from the well-mixed field sample to be processed for MPs extraction. Samples were passed through a short sieve stack (in two batches of 100 g each for 10 min on a mechanical shaker) composed of 5 mm, 1 mm, 106 μm, and 63 μm sieves to isolate the sand-sized sediment, defined here as ranging between 106 μm and 1 mm. None of the samples from either site contained particles greater than 5 mm, and only a few samples left one or two isolated shell fragments behind on the 1 mm sieve. For all samples, both at Ynyslas and at Camber Sands, the sand fraction constituted ∼99% of the sieved sediment and showed no visible organic material content.

### 2.3 Salt Solutions

We explored three different saturated salt solutions for the density separation stage: zinc bromide, sodium chloride, and calcium chloride. Each of these solutions was prepared individually and tested on independent sediment samples to assess their efficacy. Zinc bromide, as described by Quinn et al.,^22^ was initially preferred due to its ability to float all major plastic types by its high specific gravity (SG) of 1.71. The solution left a strongly brown/yellow stained coating on the membranes during the filtration stage (described further below), however, significantly reducing the ability to perform even simple visual identification, let alone FT-IR analysis. A fully saturated NaCl solution, commonly used in beach studies as a cheap and easily available density separation fluid,^23^ with a measured SG of 1.19 was notably easier to work with, but the results showed a dramatically lower MPs yields than that obtained when using a CaCl_2_ solution (as used by, for example, Stolte et al.)^24^ with a measured SG of 1.41 on the same sediment sample. The latter yielded a significantly higher number and diversity of visually recognized MPs, and CaCl_2_ was hence selected for the density separation stage.

Batches of saturated CaCl_2_ solution were prepared and passed through glass microfiber filters (1.5 μm pore size) to remove any contaminants and flocculation of salt crystals, after which the density of the solution was measured with a volumetric flask. A total of seven batches of solution were created for this study, each with a density of 1.41 (±0.01) g/cm^3^.

### 2.4 Density Separation

Each 200 (±2) g sand fraction sample was mixed with ∼300 mL of CaCl_2_ solution in a wide beaker while agitating with a glass stirrer for 2 min to create a sufficient vertical separation between settled sediment and liquid surface. As suggested by Quinn et al.^22^ a glass stirrer dipped into a surfactant (soap) was then briefly touched to the surface of the liquid to lower the surface tension that otherwise keeps afloat some smaller mineral grains, after which the solution was left to settle for 24 h. A Buchner flask connected to a vacuum line and a small length of flexible silicone tubing was then used to suction off the residue floating on top of the solution. After the top layer was collected, a glass stirrer was used to mix and agitate the sand and CaCl_2_ solution again with the aim of releasing any remaining microplastics trapped underneath the settled sediment. The solution was left to settle for another 24 h, with a second round of suctioning performed the following day. During method development, we tested an additional third round of density separation, but no further MPs were recovered; hence, two iterations appeared sufficient.

### 2.5 Vacuum Filtration

The skimmed-off supernatant (generally 50–100 mL) was diluted with 100 mL of filtered-deionized water, to help prevent salt crystallization during the filtration process, and passed through a 13 mm diameter silver membrane filter (Sterlitech) with a pore size of 5 μm, using a glass vacuum-suction filtration system. This type of membrane has not previously been used for recovering sand-sized microplastics, but rather for airborne (dust-sized) particles.^25,26^ Filtration was usually very slow (hours), and inspection under FT-IR spectroscopy showed residual salt crystallization on the filter and the particles. To eliminate this problem, silver membranes containing microplastics were placed in a small beaker and rinsed into a filtered-deionized water solution, which was then passed through a second silver membrane (at a much faster filtration rate) to be ready for analysis.

### 2.6 Identification and FT-IR Analysis

Each membrane was first inspected with optical microscopy under 10× magnification to visually map all suspected MPs based on appearance, shape, and color, following the criteria of Frias and Nash^27^ and Hidalgo-Ruz et al.^28^ For the sand-sized MPs we observed in our samples that a reliable distinction could only be made between fibers versus fragments, as we did not observe any film, foam, or pellet types. The membrane was then transferred to a Nicolet iN10 MX Fourier-Transform Infrared Imaging Microscope for chemical fingerprinting of all particles in reflective mode.^29^ For each suspected particle, spectra were acquired from an aperture window of 50 × 50 μm at three separate point locations and matched against both a polymer (OMNIC Picta, Thermo Fisher/Nicolet) and a mineral (USGS) spectral library, the match with the highest confidence being accepted. Figure 2 illustrates the imaging of suspected MPs under optical microscopy and FT-IR, as well as spectral matching. Confidence levels were recorded as low (<40%), medium (40–65%), and high (>65%). Although many studies of FT-IR spectroscopy on MPs use 70–80% as a high-confidence level, these usually involve spiking samples with virgin plastic particles.^30^ Weathering and biofouling of plastics found in the natural environment, however, degrade the purity of the spectral response, yielding less conclusive FT-IR matches.^31^ In cases of low and medium confidence, the highest matching library spectra typically did not agree on one specific polymer type but returned several different ones. The matches did, however, clearly exclude typical inorganic mineral substances such as quartz or feldspar, and together with the optical identification, this strongly suggested that the particle was a plastic regardless. Particles were classified by shape (either fiber or fragment), by plastic polymer type as listed in Table 1, and by seven color groups: yellow, blue, black, green, translucent + white, red + pink, and other (orange, purple, brown). The table shows that all of the polymer types identified among the MPs (except for rayon) have a specific gravity at or below that of the density separation solution we used (SG = 1.41) while demonstrating that a saturated NaCl solution (SG = 1.2), commonly used in other bulk studies, is not able to float (and thus fails to identify) key plastics such as polyester, PUR, PVC, and some PAs.

**Figure 2:**
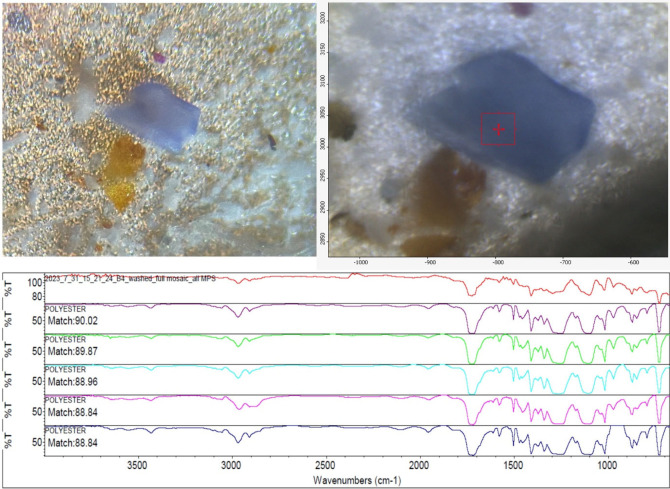
Example of MPs identification. Light blue suspected microplastic observed under a 10× optical microscope (top-left) and the same particle imaged under FT-IR microscopy (top-right), with a 50 × 50 μm spectral collection window shown with the red box with crosshairs on the center of particle; the particle is ∼0.2 mm in size. Matching of FT-IR spectra (bottom) for a pink fiber showing the collected spectrum in red at the top and five best matching spectra from libraries below; the best match in this case is polyester at 90.02%.

**Table 1:** Categories of Polymer Types Used in Recording MPs^a^

polymer type		
full name	abbreviation	specific gravity
rayon		1.53
polyester	PEST	1.40
polyethylene	PE	0.92–0.97
cellophane		1.42
nylon = polyamide	PA	1.14–1.41
polypropylene	PP	0.83–0.95
polyurethane	PUR	1.20–1.26
resin		1.11
polyvinyl chloride	PVC	1.38
ethylene vinyl alcohol	EVOH	1.12–1.24
ethylene acrylic acid copolymer	EAA	0.91–1.27
polystyrene	PS	1.01–1.10
polyethyl acrylate		1.21
acrylic = polymethyl methacrylate	PMMA	1.18

a Polymers and specific gravities adapted from Chaudhry and Sachdeva.^32^

### 2.7 QA/QC of Laboratory Protocols

The equipment used in sample processing was restricted to nonplastic materials, such as glass or metal, where practical, especially in procedures with a potential of shedding plastic fragments or fibers, such as stirring beakers or collecting sediment samples. Pure-cotton lab coats were worn, and we refrained from dry-toweling any of the glassware or equipment after washing to avoid contamination with cellulose fibers. All glassware and utensils were rinsed with ultrapure water before use, and the prepared salt solution for density separation was passed through glass microfiber filters before use (as noted in section 2.3), removing any contaminants. Density separation and vacuum filtration were conducted inside a closed laboratory fume cupboard, and membranes containing extracted MPs were transported between the fume cupboard and microscopes in closed glass Petri dishes to limit the potential of airborne contamination. The contamination risk of specifically sand-sized MPs was deemed to be low due to these mitigation measures, and we did not process blank samples, in line with many other field studies on MPs in beach sediments.^17,18,33,34^ The Supporting Information provides further details on our laboratory protocols and QA/QC measures for reliable sample analysis and mitigation of contamination, which included all those recommended by Munno et al.^35^

### 2.8 Grain-Size Analysis

The grain-size distributions of the sediment samples, after oven-drying but prior to sieving, were determined with a Malvern Mastersizer 2000 instrument, which uses laser diffraction to measure particle sizes from 0.02 μm to 2 mm in 84 logarithmically spaced bins (from ϕ = 15.6 to ϕ = −1 with intervals of 0.2), returning the % of the sample (by volume) within each size-bin. Although the Mastersizer analyses only around 3.5 g of sediment, testing of a dune sand sample with 6 replicates shows that the standard error within a size-bin is on the order of 0.2%. The grain-size distributions were analyzed with the GRADISTAT macro^36^ to return statistical parameters, such as the median grain size, *D*_50_, and moments about the mean.

### 2.9 Diversity Indices

Borrowing from the species diversity concept in ecology we calculate a normalized diversity index based on Shannon entropy to quantify a comparative diversity of microplastic particle color and plastic composition within each analyzed sample, with the normalized diversity index defined as

$$\mathrm{DI}=\frac{-\overset{R}{\underset{i=1}{\sum }}p_{i}\;\ln p_{i}}{-\ln R}$$

where *p*_*i*_ is the relative frequency of observations in class *i* (i.e., a color group or plastic type), *R* is the number of classes considered (i.e., 7 color groups or 14 plastic types), and the denominator normalizes the Shannon entropy (the numerator) by the maximum entropy attained under a perfectly uniform distribution so that the index ranges from 0 (all observations in only a single class) to 1 (observations uniformly distributed over all classes).

## 3 Results and Discussion

### 3.1 Overall Characteristics of Extracted MPs

We were able to analyze a total of 20 surface sediment samples, including both marine and aeolian deposits at both sites. For the Ynyslas site, these constituted seven beach samples, ranging from the low-tide zone to the back-beach, five dune samples, and one sample of the actively wind-blown sediment. For Camber Sands these covered two upper beach samples and five dune samples. A total of 990 sand-sized (0.1–1 mm) MPs were recorded from the optical microscopy, of which 797 were analyzed by FT-IR. Of these, 325 were chemically fingerprinted with high confidence and a further 351 at medium confidence. FT-IR analysis of two samples from Ynyslas was incomplete due to machine malfunction (57 particles unidentified for Y-DT5, 136 for Y-D2), while 10 particles were matched with high confidence to plasticine additives (specifically polyolefin, polyether, and pentaerythritol monoricinoleate) and excluded from the composition analysis.

The counts of optically identified particles from both sites combined show a mean abundance of 39 MPs in beach sand samples, with a standard deviation of 16 and a median of 47, and 35 MPs in dune sand samples, with a standard deviation of 47 and a median of 35. Fibers are the most common shape, at 87% of all detected MPs. Average fiber:fragment ratios differ between marine (10.8) and aeolian (7.3) deposits but with high standard deviations (18.2 and 4.8, respectively). Color group frequencies range from blue (33%) through black (24%), translucent + white (18%), yellow (8%), red + pink (8%), and green (5%) to other (3%). The color diversity index shows similar averages (0.78, 0.79) and standard deviations (0.12, 0.11) for marine and aeolian deposits, respectively, as does the polymer diversity index (averages 0.30, 0.32; standard deviations 0.23, 0.23). The aggregated polymer composition of the 325 high-confidence FT-IR spectral matches consists of 66% rayon, 16% polyester, and the remaining 18% spread over the other 12 polymer groups of Table 1.

Associations between color and plastic type (restricted to high-confidence matches) are explored in the color-shaded matrices of Figure 3, separated for fibers and fragments, showing that fibers predominantly consist of blue, black, and translucent/white rayon (at 22%, 18%, and 16% of all fibers, respectively), while fragments consist mostly of blue rayon (37% of all fragments). Polyester is the next most common plastic type, accounting for 15% of fibers (most commonly black) and 26% of fragments (most commonly blue). The degree of similarity between the two matrices (fibers versus fragments) was determined using the modified RV-coefficient,^37^ a correlation type measure that ranges from −1 to +1. The modified RV-coefficient for the two matrices in Figure 3 amounts to 0.86, indicating a strong similarity.

**Figure 3:**
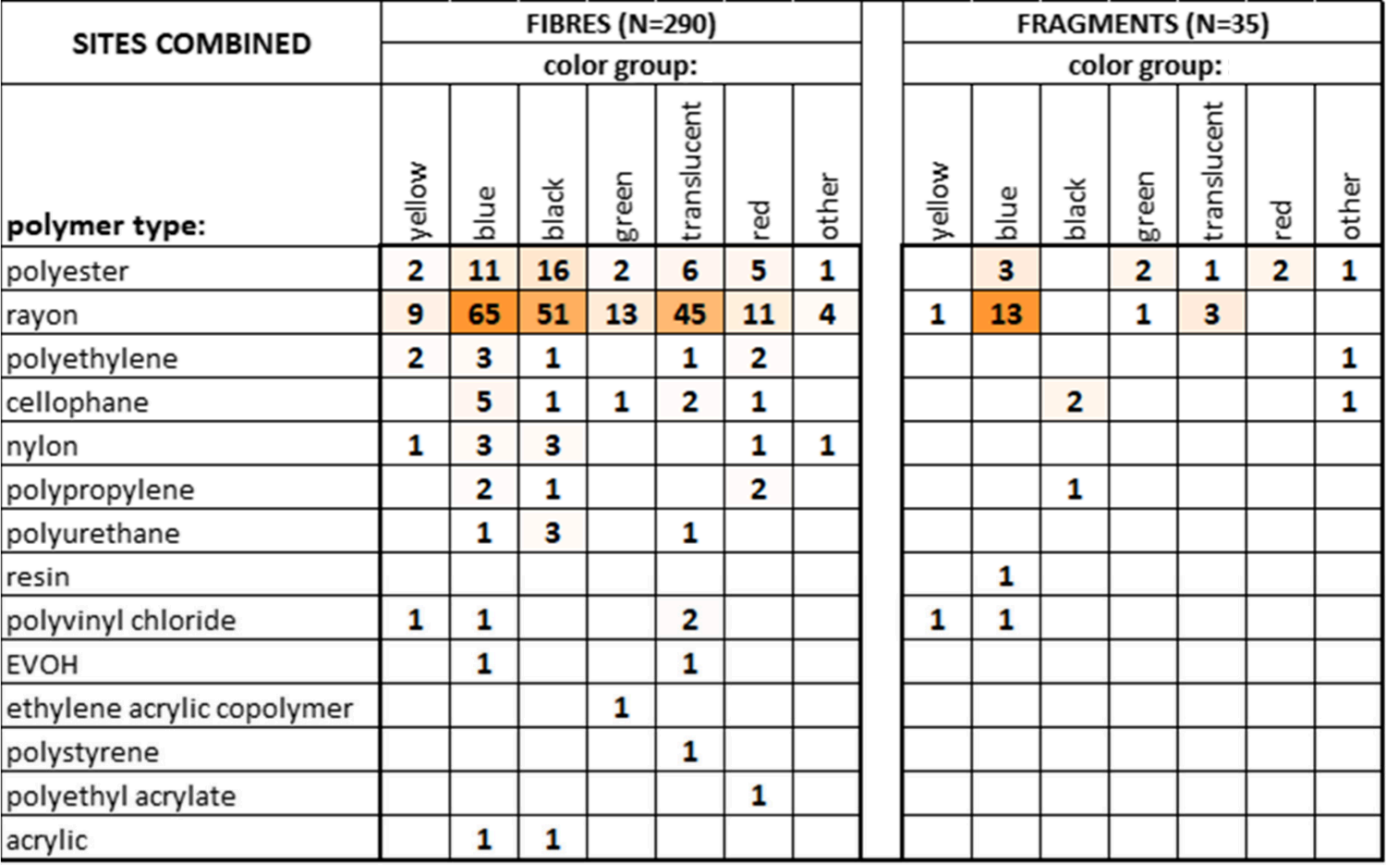
Color-shaded co-occurrence matrices showing MPs particle counts of all combinations of plastic types and color groups for identified fibers (left) and fragments (right) across all analyzed samples, from high-confidence (65+%) FT-IR identification only. Intensity of orange color-shading is proportional to the relative particle count magnitude within the individual matrix.

### 3.2 Spatial Variability and Transects

An overview of the MPs characteristics along the transects at each site is provided in Figure 4, showing total counts of particles identified under optical microscopy, counts of particles matched to plastic composition with at least medium confidence (50+%), counts for high-confidence matches only (65+%), and the two diversity indices. The results are presented along a sequence spanning from marine low-tide samples (on the left) to aeolian dune samples (on the right). A detailed breakdown with respect to each shape, color group, and plastic type of all recorded MPs is provided in the Supporting Information. Ynyslas shows an average abundance of 57 MPs per 200 g sample (marine mean = 40, aeolian mean = 78), and Camber Sands samples show a mean abundance of 35 MPs (marine mean = 37, aeolian mean = 34), with individual samples ranging from 13 to 159 MPs at Ynyslas and from 22 to 51 MPs at Camber Sands. A detailed breakdown of the results for each sample is provided in the Supporting Information.

**Figure 4:**
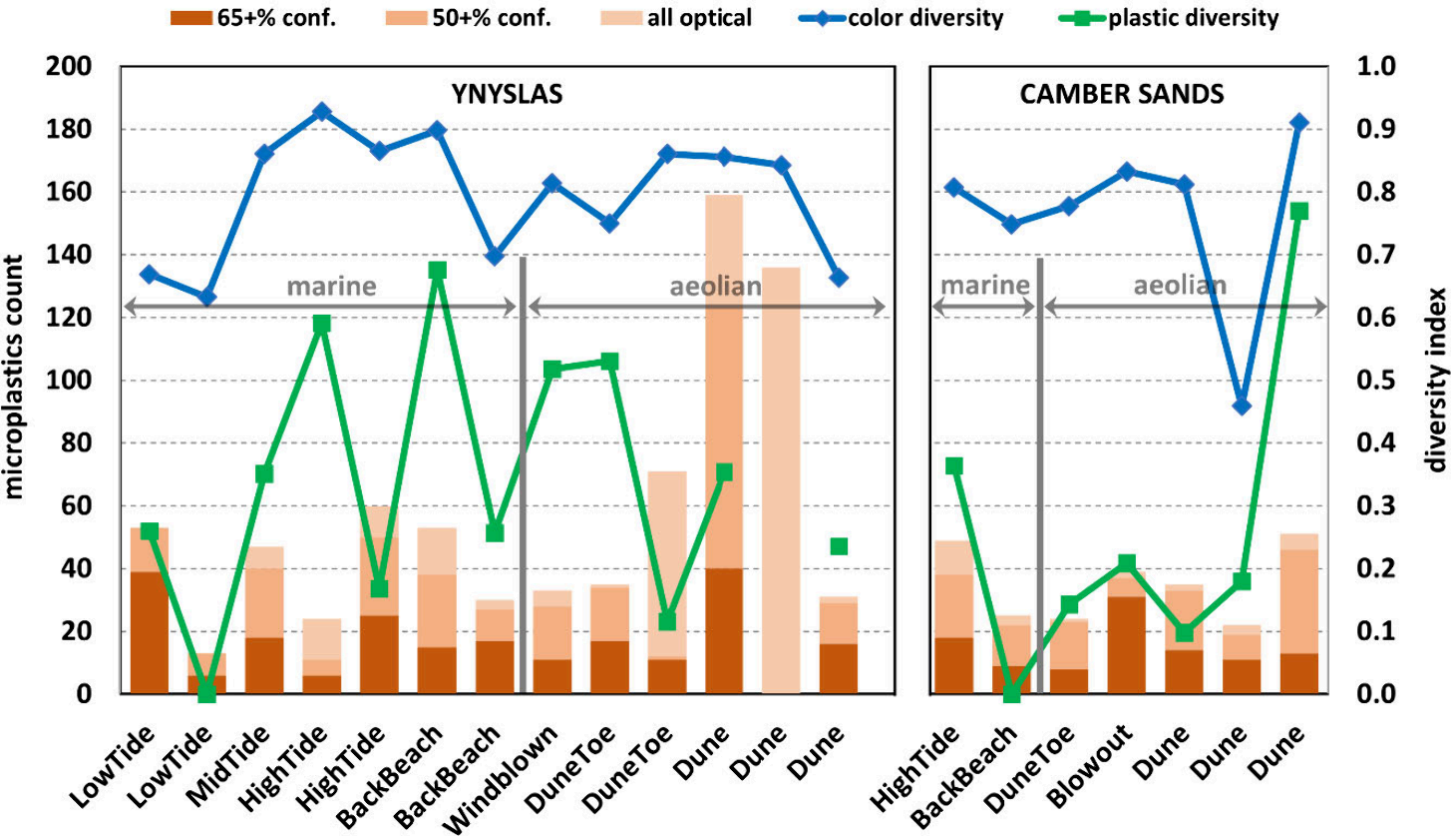
MPs counts (from 200 g samples) along cross-shore transects visualized as composite bars (cumulative) showing levels of high (65+%) confidence (dark orange), medium (50+%) + high confidence (mid orange), and all-optical (light orange) counts. Connected line graphs show magnitudes of the color diversity (in blue) and plastic diversity (in green) indices. Indicated in gray are the two sedimentary environments along each transect.

The MPs abundance shows a high spatial variability at all spatial scales: across each transect, within individual sedimentary environments, and even within like zones for all three types of particle recordings. For example, the coefficient of variation (CoV) for optically identified MPs counts is 76% for the entirety of the Ynyslas transect, with 44% among the marine and 73% among the aeolian sediment samples. Variability at Camber Sands is slightly lower, with corresponding CoV values of 34% (whole transect), 46% (marine), and 35% (aeolian). Spatial variability in color diversity is markedly lower, with CoV values of 13% and 19% for the transects at Ynyslas and Camber Sands, respectively, while variability in plastic diversity is notably high at Camber Sands (transect CoV = 101%) and less so at Ynyslas (transect CoV = 61%).

The data of counts for optically identified MPs, for FT-IR medium + high confidence, and for FT-IR high-confidence only MPs lack any sustained transect trend or pattern. Trends are similarly absent when counts from multiple samples from like zones (e.g., low-tide, mid-tide, etc.) are averaged. At Ynyslas, the two samples with very high counts either can be interpreted to suggest a trend with higher MPs counts in the dune zone or may be considered outliers compared with the other aeolian samples as well as compared with the absence of a trend at Camber Sands. An added complication is that one of these two samples (Y-D2) lacks FT-IR analysis results.

There appear to be no obvious differences in MPs polymer assemblages between marine and aeolian sediments, as shown in Figure 5 for Ynyslas (restricted to high-confidence matches). The relative frequencies of specific combinations of plastic types and color groups are similar between the two sedimentary environments and effectively mirror the fiber signatures in the aggregate (Figure 3). The modified RV-coefficient (section 3.1) for the marine versus aeolian MPs assemblage matrices at Ynyslas is 0.93, confirming a strong homogeneity of MPs in the two sedimentary environments.

**Figure 5:**
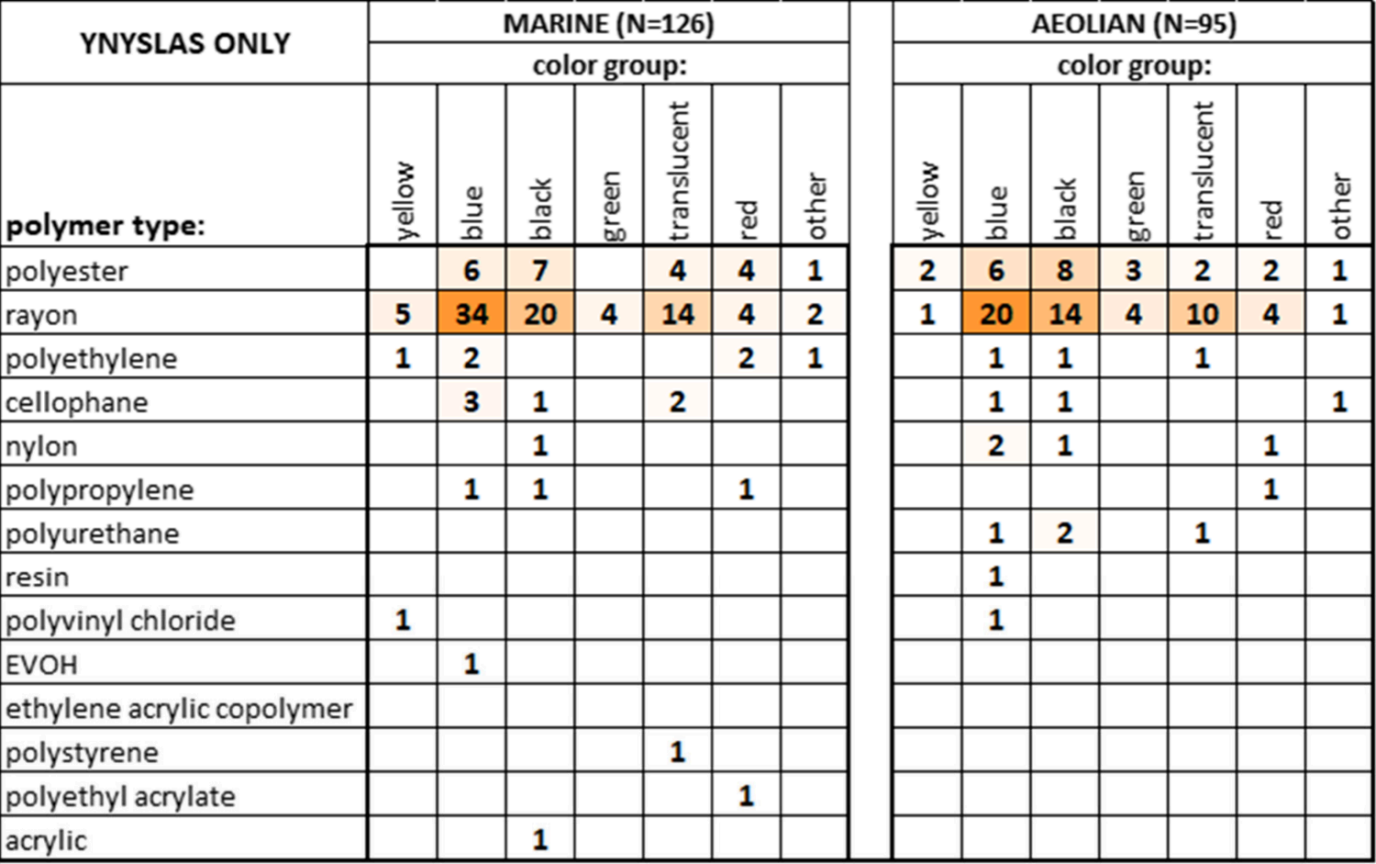
Color-shaded co-occurrence matrices showing MPs particle counts of all combinations of plastic types and color groups for beach samples (left) and dune samples (right) at Ynyslas, from high-confidence (65+%) FT-IR identification only. Intensity of orange color-shading is proportional to the relative particle count magnitude within the individual matrix.

Mann–Whitney U and Kolmogorov–Smirnov statistical tests were applied for detecting significant differences between the distributions of marine versus aeolian sediment samples for a variety of MPs parameters, including MPs counts (based on optical, FT-IR 50+% confidence, and FT-IR 65+% confidence identifications), color and plastic diversity indices, fiber abundance, and fiber:fragment ratios. All resulting *p*-values, reported in Table 2, far exceed the customary threshold of 0.05 for statistical significance (i.e., 95% confidence), indicating there are no differences in MPs abundance or diversity between these two sedimentary environments. Similar statistical testing furthermore indicates no significant difference (at 95% confidence) between Ynyslas and Camber Sands with respect to their MPs concentrations in general.

**Table 2:** Results (*p*-Values) of Two Types of Tests for Statistical Significance for Differences in the Distributions of MPs Characteristics (Independent Samples Mann–Whitney U and Kolmogorov–Smirnov) between Beach and Dune Sediment for Seven Parameters at Each of the Two Sites^a^

	Ynyslas		Camber Sands	
	M-WU	K-S	M-WU	K-S
variable tested	*p*-value		*p*-value	
counts optical MPs	0.23	0.39	0.86	0.98
counts FT-IR MPs 50+%	1.00	0.82	1.00	1.00
counts FT-IR MPs 65+%	1.00	0.97	1.00	1.00
color diversity index	0.63	0.59	0.57	0.98
plastic diversity index	1.00	0.97	1.00	0.87
counts fibers	0.37	0.40	1.00	0.98
fiber:fragment ratios	0.63	0.74	1.00	0.89

a All *p*-values greatly exceed the customary significance level of *p* < 0.05, and hence, no significant differences are detected.

### 3.3 Correlations with Sediment Grain-Size Distribution Parameters

The grain-size distributions at both Ynyslas and Camber Sands reflect unimodal, well sorted, fine sand across all samples, with an average *D*_50_ of 235 μm at Ynyslas (marine: 243; aeolian: 225) and 224 μm at Camber Sands (marine: 217; aeolian: 227). Both the *D*_50_ and the fraction of very fine sand, 63–125 μm (suggested by Anderson and Turner^18^ as a potential parameter of interest), show variability across the transects, but statistical tests (Mann–Whitney U and Kolmogorov–Smirnov) indicate no significant differences between marine and aeolian samples at each site. Comparisons at each site between these two grain-size parameters on the one hand and the number of extracted MPs on the other return very weak and effectively trivial correlations with |*r|* below 0.3 (Figure 6). Similarly, there are no meaningful correlations between MPs abundance and skewness or kurtosis either.

**Figure 6:**
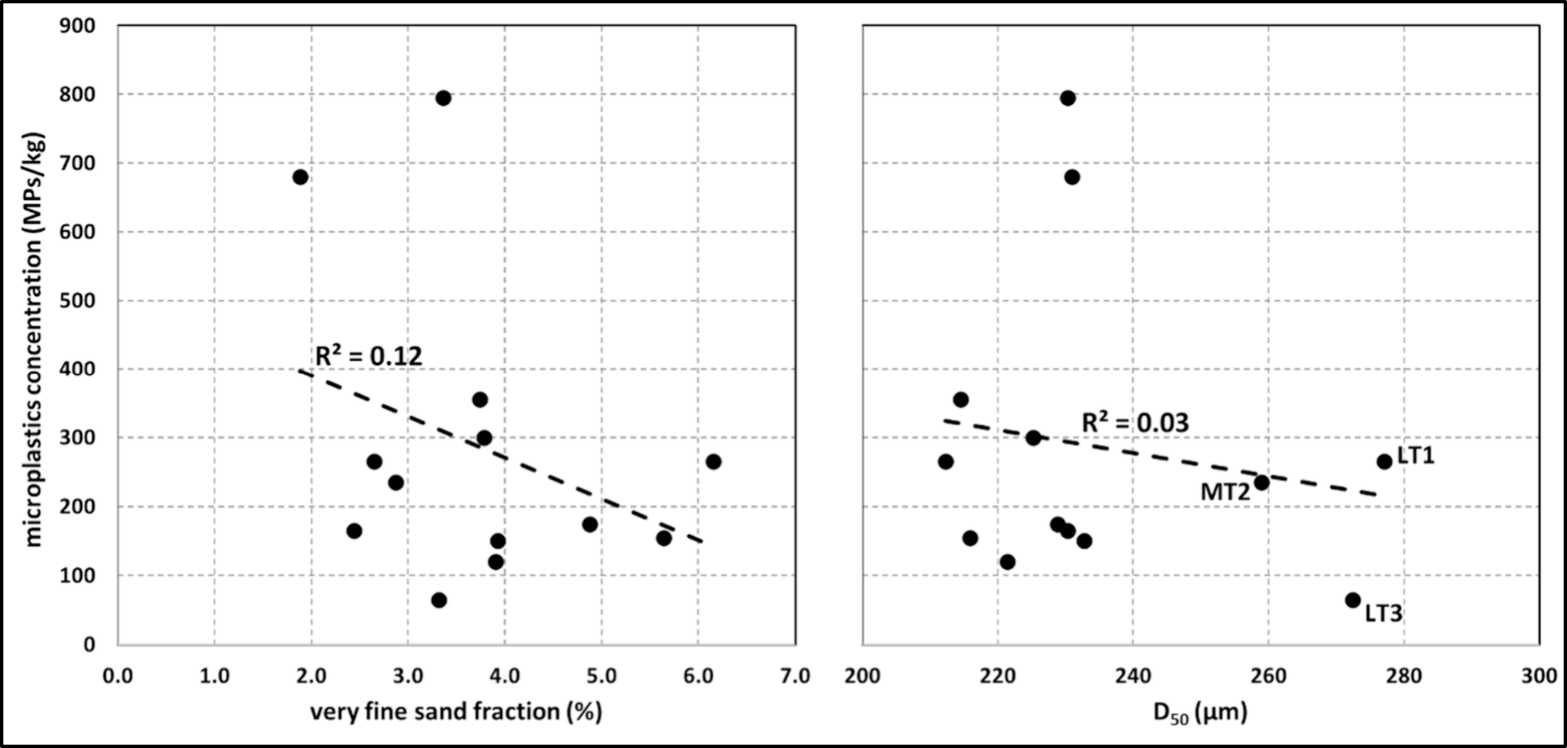
Weak and trivial correlations between grain-size distribution characteristics (very fine sand fraction left, *D*_50_ right) and MPs concentrations for the samples at Ynyslas.

### 3.4 Lessons Learned for Bulk Processing of Sediment Samples for FT-IR Analysis

Processing of 100+ grams of sediment for extracting MPs is relatively common in combination with a saturated NaCl density separation solution and filtration on large paper filters for optical microscopy analysis.^23^ We, however, aimed to use a much denser solution and much smaller filters for use in the FT-IR microscope while extracting MPs from similar amounts of sediment. There are several lessons to report. First, the purity of commercially supplied salts cannot be taken for granted, as our experience with CaCl_2_ shows: two of the three types of tested products (all from the same supplier) yielded yellow-colored solutions that stained filtered particles beyond FT-IR recognition. Second, a saturated zinc bromide solution, while promising an excellent SG of 1.7, yielded a brown flocculated liquid after mixing with the sediment, unusable for filtration. Third, passing the quantity of skimmed-off fluid containing MPs through small filters was challenging. Filters clogged up due to in situ salt crystallization under vacuum as well as the relatively high viscosity of the fluid, well before all the skimmed fluid had passed through. This required the use of multiple filters to extract all the MPs from a sample. In this process, we found that silicon membranes were too fragile and brittle to use, and we settled on the more robust silver membranes instead.

### 3.5 Relevance of Findings and Comparison with Other Studies

As the sand-sized MPs were extracted consistently from 200 (±2) g of sediment, the particle abundances found in this study translate to a mean concentration of 287 MPs/kg at Ynyslas (marine mean = 200, aeolian mean = 388) and a mean concentration of 175 MPs/kg at Camber Sands (marine mean = 185, aeolian mean = 171), with individual sample concentrations ranging from 65 to 795 MPs/kg at Ynyslas and from 110 to 255 MPs/kg at Camber Sands. In common with most other studies, these quantities strictly represent minimum abundance, as an unknown number of particles may have gone undetected or were lost during extraction^38^ and as our study excludes MPs smaller than 0.1 mm. The above concentrations in the beach sediments are comparable in order of magnitude to many other studies of beach surfaces, both in the UK^18,34^ and globally,^39–45^ although exact comparisons between studies are confounded by differences in methods (especially field sampling, density separation, and detection) as well as the MPs size fractions extracted. Although no statistically significant difference was found between the two sites, the concentrations at Ynyslas are on average higher than those at Camber Sands, which is inconsistent with the much more intense beach tourism at the latter site, suggesting that MPs sourcing from the marine reservoir (rather than in situ from locally generated surface waste) is more important, as also found previously by Ronda et al.^46^

The composition of the MPs in the beach surface sediment is consistent with those of many other studies in the coastal zone. The dominance of fibers has been observed by Doyen et al.^47^ on French beaches along the English Channel and by Lots et al.^41^ at many other European beaches. The high fiber concentration we find at Ynyslas and Camber Sands may be associated with the numerous sewage spills that regularly occur in Cardigan Bay^48^ and the English Channel,^49^ which likely include a large amount of washing machine runoff containing wear and tear from synthetic consumer textiles. The most commonly detected compound by far in both study areas is rayon, followed by polyester. Although the latter is undeniably a plastic material, rayon comes with some ambiguity. Rayon is a synthetic material typically originating from natural (nonplastic) fiber sources such as cellulose, which then undergoes drastic chemical processing where (polymer) dyes, additives, and stabilizers are added to achieve desired fabric qualities.^50^ Because its negative chemical effects upon breakdown and ingestion are similar to those of nylon and polyester, rayon fibers are typically classed as MPs.^51^

For MPs in coastal dune sediments, our results can be compared with the two previous studies reported in the literature so far. Costello and Ebert^17^ found up to 12 MPs per m^2^ surface area in coastal dunes around Lake Erie and Lake Ontario. Since the MPs in our 200 g analysis are ultimately derived from ∼700 g of sediment sampled from 0.12 m^2^, the mean abundance in aeolian surface samples at Ynyslas and Camber Sands extrapolates to 2260 and 998 MPs/m^2^, respectively, both 2 orders of magnitude greater than the Costello and Ebert results (although sampling depths are slightly different). Our results are very similar, however, to the concentrations found in the Braunton Burrows dunes by Anderson and Turner,^18^ who report approximately 180–575 MPs/kg. Unlike that study, though, we find no correlation between the MPs concentrations and grain-size distribution parameters, partly perhaps because of the much smaller range of very fine sand fractions (2–6% at Ynyslas, compared with 0–14% at Braunton) and the lack of any *D*_50_ differentiation.

The key findings of our study are the absence of any significant differences in sand-sized MPs concentrations, as well as their polymer and color diversity, in beach (marine) versus dune (aeolian) surface sediments, as well as the homogeneity of the polymer assemblages of the MPs in these sedimentary environments. The similarity with respect to MPs concentrations was also found by Anderson and Turner,^18^ whereas the homogeneity with respect to polymer composition and diversity is a novel insight. These findings suggest that there is no MPs enrichment of the coastal dunes in terms of number, shape, or polymer type.

Our study distinguished only between fibers and fragments, as we did not observe other shapes such as foams, films, or pellets. This is consistent with other studies of wind-blown MPs that only report fibers and fragments (e.g., Wang et al.)^52^ and is most likely related to the 0.1–1 mm (sand) size range of the MPs we extracted, since more distinctive shapes like foams and pellets appear to be most commonly found (or identifiable) at sizes above 1 mm.^17,33^

The total number of MPs detected in this study (*N* = 990) and particularly the number of particles analyzed for polymer composition (*N* = 797, of which 676 were identified at medium-to-high confidence) have, to our knowledge, not been achieved in previous comparable studies in the coastal zone.

### 3.6 MPs Homogeneity of Marine and Aeolian Surface Sediments: New Hypotheses

The lack of MPs enrichment of coastal dune sediment is at odds with the wind tunnel studies^13,14^ reporting highly preferential aeolian transport of MPs (particularly fibers) relative to mineral sand. MPs concentrations in the beach sediment are twice-daily replenished from the marine reservoir under the tidal cycle, and so preferential aeolian transport of MPs from the beach into the coastal dunes ought to result in a relative increase of concentrations in aeolian deposits, particularly for fibers. Our results, however, are more consistent with the findings of Tian et al.^15,16^ showing less evidence of preferential transport of MPs.

Several hypotheses may be advanced for the lack of coastal dune enrichment. First, MPs may be confined primarily to surface sediment so that they effectively “wash” through the aeolian sedimentary system and either are exported out of the coastal dunes (suggested by Anderson and Turner)^18^ or only accumulate further downwind of the foredunes which have been sampled in studies so far. In our field work we collected sediment from areas on the foredune that had visibly accumulated sand (as evidenced by partially buried marram grass, for example), but it is possible that MPs, especially fibers, effectively remain “afloat” on the surface in a net-depositional saltation environment. The sample we collected from an erosional surface on the side of a blowout at Camber Sands (CS-BL1), however, does not show a particularly low MPs abundance, contradicting this model. This hypothesis may be tested in the future by collecting depth samples and longer transects within coastal dunes.

Second, the very high spatial variability in MPs abundance may preclude the detection of relative enrichment in the dune sediment, i.e., the variability in concentrations is higher than the effect of preferential transport. This begs the question of why MPs concentrations are so variable within the surface sediment in both marine and aeolian environments. In our study the MPs counts are ultimately derived from ∼700 g of original field sample sediment, equivalent to 440 cc (assuming a bulk density of 1.6 g/cm^3^), extracted from a 0.12 m^2^ surface area. Individual sampling locations within a transect zone were generally several meters apart, suggesting that MPs concentrations vary on a spatial scale of at least meters. The reported enrichment ratio for fibers of up to 1000 in the Bullard et al.^14^ wind tunnel study ought to be sufficient to overcome the masking effect of spatial variability, however. The transect at Ynyslas displays a standard deviation in fiber counts of 41 compared with a mean of 50 (CoV = 82%), and so an enrichment of microplastic fibers in the aeolian sediments on the order of 1000 should have easily exceeded this general level of variability. This hypothesis may be tested in the future by analyzing a much greater number of replicates in each sedimentary zone.

Third, and we believe most likely, the aeolian transfer of MPs from the beach surface into the coastal dunes is severely supply-limited, whereas the movement of the mineral sand matrix is mainly transport-limited. That is, even though MPs can much more easily be moved by wind, there are so few particles available at the surface that their maximum possible transport rate remains at a very low magnitude and their supply is furthermore very quickly depleted, whereas the flux of sand grains increases nonlinearly with wind speed, from an unconstrained supply, and reaches rates many orders of magnitude higher. Due to the very limited supply in the source sediment, any significant MPs enrichment of the flux, compared with mineral sand, can only be reached within a very narrow range of wind speeds (above the threshold for the motion of MPs but well below that for sand grains) because once the wind starts to move even just a small fraction of available sand grains, the enrichment differentiation is lost. Wind at a speed of just 1% above the threshold for initiation-of-motion at Ynyslas, for example, already generates a sand flux on the order of 10^5^ grains m^–1^ s^–1^ (based on standard sand transport models),^53^ dwarfing the number of available MPs. Natural winds that transport sediment from the beach surface into the coastal dunes fluctuate over a wide range of speeds and most of the time greatly exceed the narrow wind speed range required for detectable MPs enrichment in the flux. The severe MPs supply limitation is illustrated by the consideration that an idealized perfectly smooth 1 m^2^ of sediment surface composed of one layer of sand grains constitutes on the order of 10^7^ particles, 4 orders of magnitude more than the 1000 to 2000 MPs/m^2^ found in our study. The absence of MPs enrichment in the sediment flux is corroborated by the results from the wind-blown sample collected at Ynyslas (Y-W2), which shows no obviously elevated level of MPs concentration relative to the beach surface. This hypothesis may be tested in wind tunnel experiments by using lower MPs concentrations of only hundreds of MP/kg as found on sandy beaches, as well as by measuring the number of MPs truly exposed and available for aeolian transport at the air–sediment interface.

### 3.7 Implications and Future Research

Our findings suggest that monitoring of MPs in beach sediment may be sufficient for representing the contamination of the terrestrial coastal zone since the composition of the MPs assemblage is similar across both the intertidal beach and the coastal dunes. Furthermore, as the coastal dunes are not enriched in MPs but only reflect the deposition of supply-limited MPs blown in from the beach, any potential mitigation efforts may focus on the marine environment as a priority.

Future field research should explore sampling strategies for reducing the impact of spatial variability in MPs contamination and/or identifying its origins. More significant advancement of our understanding of wind-blown transport of MPs (particularly fibers) in the coastal zone requires experimental work, wind tunnel studies, and numerical simulations representing real-world contamination levels where the number of MPs exposed to potential entrainment by wind at the sediment surface is very small (by 4 orders of magnitude) relative to the number of ordinary mineral sand grains.

## Supplementary Material



## Notes

Laboratory protocols and QA/QC for reliable sample analysis and mitigation of contamination during the MPs extraction and identification; inventory of equipment used; and detailed breakdown of MPs counts and polymer identification results for each sample (PDF)
