# Supplementary material for: Homogeneity of Sand-Sized Microplastics Concentration and Polymer Assemblage in Beach and Coastal Dune Sediments
Source: Environ Sci Technol. 2025 Oct 28;59(44):24012–22. doi: 10.1021/acs.est.5c09174 (PMC12613819; doi:10.1021/acs.est.5c09174)
Supplement: Supplementary file 1 [file es5c09174_si_001.pdf]

SUPPORTING INFORMATION

Homogeneity of sand-sized microplastics concentration and polymer assemblage in beach and coastal dune sediments

Rasma Ormane[1] and **Andreas CW Baas**[1]*

[1]: Department of Geography, King's College London, London, UK

*: corresponding author: andreas.baas@kcl.ac.uk

4 pages, including two tables and:

- Laboratory protocols and QA/QC for reliable sample analysis and mitigation of contamination
- Detailed breakdown of MPs counts and polymer identification for each sample

Laboratory protocols and QA/QC for reliable sample analysis and mitigation of contamination (supplementing section 2.7. 'Limiting contamination')

All laboratory work was conducted in the JB Thornes Earth Surface Materials lab of the Geography Department at King's College London, providing a multi-room CL2 laboratory environment that includes a dedicated internal airflow design that separates spaces reserved for relatively 'clean' wet-lab chemistry (upstream) from spaces where work may generate surface and air particulates (downstream). It is worth reiterating here that the study extracted and analysed MPs in the sand-size range from 0.1 to 1.0 mm. QA/QC hence did not concern particles much smaller than 100 µm.

Equipment used in the collection, storage, and processing of samples was restricted to non-plastic materials, such as glass or metal, where practical, especially in procedures with a potential of shedding plastic fragments or fibres, such as stirring beakers or collecting sediment samples. **Table SI1** below, lists a full inventory of all items and materials used. Only a few plastic items were unavoidable, and if a suspected MP particle was matched against any of these exact polymer types and colours during the FTIR analysis the particle was not recorded as an original MP.

Table SI1: *Inventory of equipment used and the materials they were made of.*

Sample stage	Equipment	Material	Mitigation
Collection	sampling quadrat	steel	
	spoon	steel	
	containers	aluminum	
Drying	containers	aluminum	
Sieving	sieve shaker	steel and rubber	
	mesh sieves	steel	
Salt solution	calcium chloride	PFHD containers	salt solution was filtered before use
	hotplate stirrer	steel	
	magnetic stirrer bar	PTFE (polytretrafluoroethylene)	
	volumetric flask	glass	
	funnel	glass	
	filters	paper/glass microfibre	
	cover	aluminum foil	
	nitrile gloves	synthetic rubber	
Density separation & Vacuum filtration	beaker	glass	
	buchner flask	glass	
	stirring rod	glass	
	suction tubing	silicone	
	wash bottle	LDPE (low-density polyethylene)	MPs excluded
	filtration system	glass	
	membrane filter	silver	
	tweezer	steel	
	cover	aluminum foil	
Microscopy	microscope slide	glass	
	petri dish+lid	glass	

The laboratory protocol followed all relevant measures recommended by Munno *et al.* (2023) to ensure reliable sample analysis and mitigation of contamination by external MPs during processing and analysis:

- All wet-lab procedures were conducted inside a closed low-flow fume cupboard (Munno *et al.* recommendation #1) and samples were kept covered with aluminum foil when not worked on (recommendation #2) to minimise airborne contamination. Similarly, silver membranes with MPs deposits were transferred across the lab space inside closed glass petri-dishes.
- All wet-lab equipment was triple-rinsed using ultra-pure (Type I) water – produced by an ELGA PURELAB Option-Q system – before each sample was processed (recommendation #2).
- Pure-cotton lab coats were worn by the authors and lab staff at all times and polymer-based clothing was avoided (recommendation #3).
- The salt solution for density separation was filtered prior to use, through Whatman Grade 1 paper filters in the early part and through glass microfibre filters in the latter part of the work, to remove any contaminants (recommendation #4).
- All wet-lab work was conducted only by the two authors to ensure consistency and all FT-IR identification work was conducted solely by RO to ensure any subjective bias was consistent across all samples (recommendation #5).
- Handling of samples was minimised by a systematic processing sequence and short transfer distances (recommendation #6).

Methods development and blanks

Several findings during the preliminary methods development supported QA/QC of the laboratory protocol:

- Test processing of field samples revealed apparent contamination with blue cellulose fibres originating from paper towel rolls that had been used for drying of glassware. Hence, we refrained from dry-towelling any of the glassware or equipment after washing.
- Although we did not include procedural blanks during the mature stage of the work, blanks analysis was part of the methods development. Several tests involved passing prepared salt solution through the silver membrane filtration procedure (including usage of the pertinent glassware and equipment) and subsequent inspection under the optical microscope did not expose any sand-sized MPs.
- Testing of the density separation procedure on field samples showed that a third round of sediment agitation in the salt solution (followed by 24 hours of settling) did not yield any newly released sand-sized MPs when investigating the filtered supernatant under the optical microscope. This suggests there was little scope for contamination by external MPs during the filtration and microscopy procedures.

Munno, K., Lusher, A.L., Minor, E.C., Gray, A., Ho, K., Hankett, J., Lee, C.F.T., Primpke, S., McNeish, R.E., Wong, C.S. and Rochman, C., 2023. Patterns of microparticles in blank samples: A study to inform best practices for microplastic analysis. *Chemosphere*, *333*, p.138883.

***Table SI2*: Detailed breakdown of MPs counts and polymer identification for each sample**

(supplementing section 3.2. 'Spatial variability & transects')

	YNYSLAS													CAMBER SANDS							Ynyslas	Camber Sands	aggregate
sample code	Y-LT1	Y-LT3	Y-MT2	Y-HT2	Y-HT3	Y-BB2	Y-BB4	Y-W2	Y-DT1	Y-DT5	Y-D1	Y-D2	Y-D4	CS-HT1	CS-BB1	CS-DT1	CS-BL1	CS-D1	CS-D2	CS-D4			
sedimentary environment	marine	marine	marine	marine	marine	marine	marine	aeolian	aeolian	aeolian	aeolian	aeolian	aeolian	marine	marine	aeolian	aeolian	aeolian	aeolian	aeolian			
cross-shore zone	LowTide	LowTide	MidTide	HighTide	HighTide	BackBeach	BackBeach	Windblown	DuneToe	DuneToe	Dune	Dune	Dune	HighTide	BackBeach	DuneToe	Blowout	Dune	Dune	Dune			
optical MPs	53	13	47	24	60	53	30	33	35	71	159	136	31	49	25	24	39	35	22	51	745	245	990
FT-IR MPs 50^{+}%	53	13	40	11	50	38	27	28	34	*12*	123		29	38	22	23	37	33	19	46	458	218	676
FT-IR MPs 65^{+}%	39	6	18	6	25	15	17	11	17	*11*	40		16	18	9	8	31	14	11	13	221	104	325
colour diversity index	0.67	0.63	0.86	0.93	0.87	0.90	0.70	0.81	0.75	0.86	0.86	0.84	0.66	0.81	0.75	0.78	0.83	0.81	0.46	0.91	0.87	0.85	0.87
plastic diversity index	0.26	0.00	0.35	0.59	0.17	0.68	0.26	0.52	0.53	*0.12*	0.35		0.24	0.36	0.00	0.14	0.21	0.10	0.18	0.77	0.48	0.40	0.47
**SHAPE**	**SHAPE**													**SHAPE**							**SHAPE**		
fibre	43	6	40	21	59	44	26	25	33	62	148	125	24	38	21	19	39	28	18	44	656	207	863
fragment	10	7	7	3	1	9	4	8	2	9	11	11	7	11	4	5		7	4	7	89	38	127
**COLOUR**	**COLOUR**													**COLOUR**							**COLOUR**		
yellow	2	3	3	5	6	5	1	4	1	7	9	10	1	4	1	1	1	4	2	8	57	21	78
blue	22	6	14	7	20	20	14	12	8	27	57	26	18	15	11	6	13	13	7	14	251	79	330
black	20		9	3	15	8	6	9	15	12	37	45	4	17	4	5	8	8		8	183	50	233
green	1		4	2	4	3			1	3	8	3	3	2	2	2	5	1		7	32	19	51
translucent (+white)	6	3	7	3	5	4	7	3	5	14	29	36	1	8	6	9	7	7	13	10	123	60	183
red (+pink)	2		10	3	9	6	1	3	5	4	11	10	4	2	1	1	5	1		3	68	13	81
other (orange, purple, brown)		1	0	1	1	7	1	2		4	8	6		1				1		1	31	3	34
**PLASTIC (high-confidence)**	**PLASTIC (high-confidence)**													**PLASTIC (high-confidence)**							**PLASTIC (high-confidence)**		
polyester	6	6	4	1		2	3	1	5		7		11	1				1		4	46	6	52
rayon	30		12	2	22	4	13	5	6	*10*	28		5	13	9	7	26	13	9	2	137	79	216
polyethylene					2	4			1	*1*	1									1	9	1	10
cellophane	3		1			1	1	3						2			2				9	4	13
nylon						1			1		3						3			1	5	4	9
polypropylene			1			2		1						1						1	4	2	6
polyurethane									4					1							4	1	5
resin											1										1		1
polyvinyl chloride				1				1								1			2	1	2	4	6
EVOH						1														1	1	1	2
ethylene acrylic copolymer																				1		1	1
polystyrene				1																	1		1
polyethyl acrylate				1																	1		1
acrylic					1															1	1	1	2
